# PI3Kγ Is Critical for Dendritic Cell-Mediated CD8+ T Cell Priming and Viral Clearance during Influenza Virus Infection

**DOI:** 10.1371/journal.ppat.1005508

**Published:** 2016-03-31

**Authors:** Samuel Philip Nobs, Christoph Schneider, Alex Kaspar Heer, Jatta Huotari, Ari Helenius, Manfred Kopf

**Affiliations:** 1 Molecular Biomedicine, Institute of Molecular Health Sciences, Department of Biology, ETH Zurich, Zurich, Switzerland; 2 Institute of Biochemistry, ETH Zürich, Zurich, Switzerland; Erasmus Medical Center, NETHERLANDS

## Abstract

Phosphoinositide-3-kinases have been shown to be involved in influenza virus pathogenesis. They are targeted directly by virus proteins and are essential for efficient viral replication in infected lung epithelial cells. However, to date the role of PI3K signaling in influenza infection in vivo has not been thoroughly addressed. Here we show that one of the PI3K subunits, p110γ, is in fact critically required for mediating the host’s antiviral response. PI3Kγ deficient animals exhibit a delayed viral clearance and increased morbidity during respiratory infection with influenza virus. We demonstrate that p110γ is required for the generation and maintenance of potent antiviral CD8+ T cell responses through the developmental regulation of pulmonary cross-presenting CD103+ dendritic cells under homeostatic and inflammatory conditions. The defect in lung dendritic cells leads to deficient CD8+ T cell priming, which is associated with higher viral titers and more severe disease course during the infection. We thus identify PI3Kγ as a novel key host protective factor in influenza virus infection and shed light on an unappreciated layer of complexity concerning the role of PI3K signaling in this context.

## Author Summary

Acute respiratory viral infections like influenza virus can cause life-threatening disease in infected individuals. Phosphoinositide-3-kinases have been suggested to be important factors used by the virus to infect and replicate in host cells, and thereby cause viral pneumonia. However, to date the role of these signaling molecules has not been thoroughly addressed in the context of an infection in whole animals, rather than just cell culture systems. Here we show that one of the PI3K subunits, PI3Kγ, is in fact critically required for the clearance of the infection. This is because PI3Kγ regulates the immune response against the virus through the generation and maintenance of antiviral CD8+ T cell responses. We show that in the absence of PI3Kγ a specialized dendritic cell subset in the lung is deficient and this leads to a strongly impaired immune response against influenza virus. We thus identify PI3Kγ as a novel host molecule that is important for the immune defense against influenza virus infection

## Introduction

Phosphoinositide 3-kinases (PI3K) are classified into three main groups (class I, class II and class III) according to sequence homology of the catalytic subunit and their substrate specificity [1]. Class I PI3K are further divided into class IA and class IB. Class IA PI3K form dimers consisting of either one of the catalytic subunits p110α, p110β or p110δ, and the common regulatory subunit p85 [2] [3] [4] [5]. They typically act downstream of receptor tyrosine kinases and are important regulators of cell growth, division and survival [6]. In contrast, class IB PI3K (also termed PI3Kγ) comprises only one catalytic subunit, p110γ, which associates with the regulatory subunits p101 or p84 [7] [8] [9] [10] [11]. PI3Kγ signals downstream of G-protein coupled receptors (GPCR) such as chemokine receptors or receptor tyrosine kinases [12]. Both class IA and PI3Kγ can be activated by ras [13] [14]. Classes II and III PI3K are ubiquitously expressed and mainly involved in regulation of protein trafficking and cell homeostasis. PI3Kγ on the other hand is preferentially expressed in hematopoietic cells, although expression was also shown in peribronchial epithelial cells, the endothelium, the brain and the heart [15] [16].

Several groups have addressed the role of PI3Kγ in immune responses using specific inhibitors or p110γ-deficient mice. Neutrophils and macrophages, which are p110γ-deficient, exhibit reduced migration *in vitro* in response to chemotactic stimuli such as IL-8 and MIP-1α as well as the GPCR agonists C5a and fMLP [17]. Consistently, *in vivo* recruitment of neutrophils and macrophages to inflamed peritoneum is severely impaired in p110γ-/- animals upon peritoneal infection with *Listeria monocytogenes [18]*. In addition to the defects observed in innate immune cells, PI3Kγ-deficiency results in impaired adaptive immune responses. PI3Kγ-signaling in conjunction with PI3Kδ, plays a minor role in thymocyte as well as B cell development and the absence of PI3Kγ leads to a small reduction of peripheral CD4+ but not CD8+ T cells [19]. Addressing the migration capacity of lymphocytes, it was shown that PI3Kγ is superfluous for T cell homing in steady-state conditions [20]. Under inflammatory conditions however, PI3Kγ-/- mice display a reduced recruitment of CD8+ T cells. Peritoneal infection with Vaccinia virus or Lymphocytic Choriomeningitis virus infection into the footpads result in decreased numbers of CD8+ T cells at the site of inflammation in PI3Kγ-/- mice [21] [22]. In both studies, PI3Kγ-/- CD8+ T cells exert normal effector functions in terms of Interferon-γ (IFN-γ) production and cytoxicity. In contrast to T cells, B cells develop normally in PI3Kγ-/- mice and do not show any deficiency in migration [20]. More recently, we could show that PI3Kγ is required for development of lung CD11b+ DC and CD103+ DCs in particular by regulation of signaling downstream of Flt3, while it is dispensable for DC development in many other tissues [23]. In line with this data, several reports have revealed a central role for PI3Kγ in murine models of human immune-mediated inflammatory diseases such as rheumatoid arthritis and airway inflammation as well as autoimmune diseases such as systemic lupus [24] [25] [26]. Therefore, PI3Kγ is considered a promising target for the treatment of inflammatory disorders [27].

Using chemical inhibitors such as Wortmannin, PI3K family members or effectors downstream such as Akt kinases were shown to be required by influenza virus for infection of lung epithelial cells *in vitro* [28] [29], in particular through interactions with the viral protein NS1 [30]. Furthermore, Influenza virus strains carrying mutations rendering them unable to activate PI3K signaling were shown to lead to attenuated infection *in vitro* and *in vivo* [30]. However, the importance of PI3K signaling for host defense as well as the specific roles of individual PI3K subunits for influenza virus infection *in vivo*, remain poorly understood. In this context PI3Kγ itself has not received much attention in the context of influenza infection. Given the defects in innate and adaptive immunity in PI3Kγ-deficient mice and its potential direct involvement in influenza virus pathogenesis, we investigated the role of PI3Kγ-signaling upon infection with influenza virus *in vivo*. We found that PI3Kγ-deficiency led to greatly enhanced susceptibility to influenza virus infection due to delayed viral clearance. This was caused by impaired T cell priming by lung resident dendritic cells due to a pre-existing developmental deficiency in the lung dendritic cell compartment of PI3Kγ-deficient animals. We thus describe a novel role of PI3Kγ in regulating host responses against respiratory viral infections.

## Results

### p110γ-KD mice are highly susceptible to influenza virus infection

**Fig 1:**
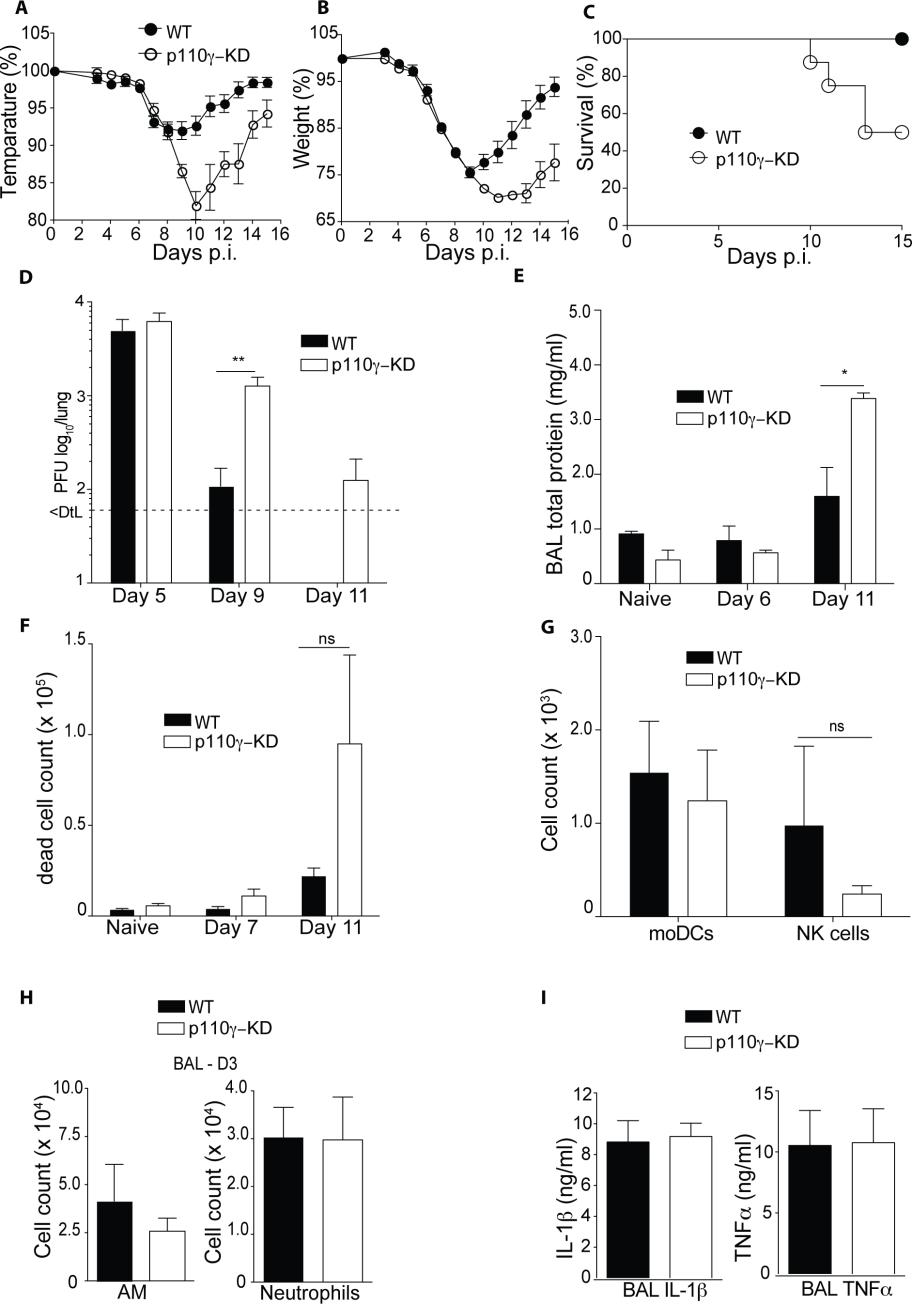
p110γ-deficiency is associated with severe disease and delayed viral clearance during influenza virus infection. WT and p110γ-KD mice were infected with 50 pfu PR8 IAV, shown are (A) temperature and (B) weight, relative to the day of infection (n = 8). WT and p110γ-KD mice were infected with 200 pfu, shown is(C) a survival curve showing the fraction of surviving animals for each day p.i (n = 10). WT and p110γ-KD mice were infected with 50 pfu PR8 IAV, shown are (D) lung viral titers (n = 5), (E) BAL total protein content (n = 5) and (F) number of eFluor780+ dead cells in the BAL of infected animals at different time-points (n = 5). At day 3 p.i. the immune cell infilitrate in the BAL was quantified, all cells were pregated on CD45+ viable cells, shown are (G) moDCs identified as CD11c+MHCII+CD11b+Ly-6C+ cells, NK cells identified as CD49b+CD3-NK1.1+ cells, (H) AM, identified as Siglec-F+ CD11c+ cells and neutrophils as CD11b+Ly-6G+ cells (n = 4) (I) At day 3 p.i. the cytokine levels of TNFα and IL-1β in the BAL was quantified using ELISA (n = 4). For all bar graphs mean ± SEM is shown and results are representative of at least 2 experiments. The Student’s t test (unpaired) was used: p < 0.05 (*), p < 0.01 (**), p < 0.001 (***), p < 0.0001 (****).

To address the role of p110γ in Influenza A virus infection (IAV) *in vivo* we infected p110γ kinase–dead (p110γ-KD) animals with a sub-lethal dose of the highly pathogenic strain IAV PR8. These animals carry an inactivating mutation in the kinase domain of p110γ and thus allow us to delineate the role of p110γ kinase function during IAV infection *in vivo*. Monitoring weight and temperature loss over time, we observed a much more severe disease course in p110γ-KD animals as opposed to WT controls with a more pronounced temperature and weight loss (Fig 1A and 1B). Furthermore, at a fourfold higher dose (i.e. 200 pfu), half of the p110γ-KD mice succumbed to infection by day 15, while 100% of WT animals survived (Fig 1C). The enhanced morbidity observed in p110γ-KD mice infected with 50 pfu was paralleled by a delayed viral clearance at later time-points of infection, where p110γ-KD animals had higher viral titers at day 9 and 11 p.i, while there was no difference at day 5 p.i (Fig 1D). In addition, the high viral load in lungs of p110γ-KD animals at later points during the infection correlated with an increased proteinosis and cell death in the alveoli, exemplified by higher levels of total protein and higher number of dead cells in the bronchoalveolar lavage (BAL) of p110γ-KD mice at day 11 (Fig 1E and 1F). Examining the immune cell infiltrate in the lungs of infected animals at an early time point of infection it was apparent that recruitment of monocyte-derived dendritic cells (moDC) natural killer (NK) cells and neutrophils was completely intact despite p110γ kinase-deficiency (Fig 1G and 1H). Similarly, numbers of tissue-resident alveolar macrophages (AM) were comparable between WT and p110γ-KD animals at day 3 p.i. (Fig 1H). Finally, also levels of hallmark inflammatory cytokines TNFα and IL-1β were similar in the BAL of both mouse strains (Fig 1I). Taken together these results suggested that p110γ plays an important role in host defense against IAV but that the early antiviral response is largely intact.

### p110γ-KD mice exhibit a strongly impaired antiviral CD8+ T cell response against influenza virus

**Fig 2:**
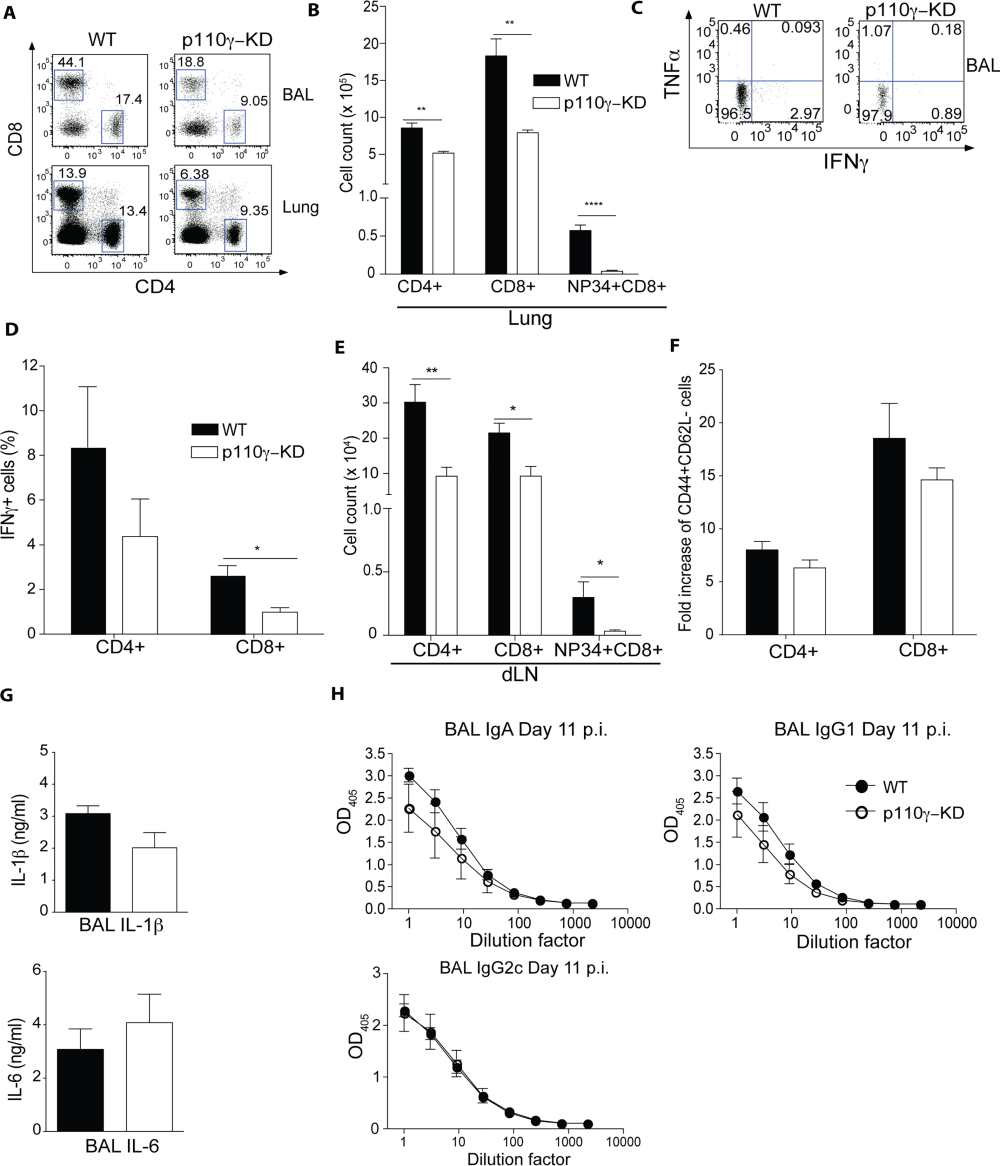
p110γ is required for the antiviral CD8+ T cell response. WT and p110γ-KD mice were infected with 50 pfu PR8 IAV and the T cell response was analysed at day 7 p.i. Cells were pregated on CD45+ viable cells. Shown are (A-B) representative dot plots of CD4+ and CD8+ T cells in the BAL and lungs of infected animals as well as (B) a summary of the lungs of all mice including NP34 Tet+ virus-specific CD8+ T cells (mean ± SEM) (n = 5) (C-D) BAL cells from infected animals were restimulated with NP34 peptide and inactivated PR8 IAV, shown are representative dot plots as a well as a summary of all animals (n = 5) (mean ± SEM). (E) Shown is a quantification of T cells of the lung dLN of all analysed mice (mean ± SEM)(F) Activated T cells were identified as CD44+CD62L-. Shown are the fold increase in total numbers compared to naive lungs (G) Shown is a summary of the cytokine levels in the BAL of infected animals (mean ± SEM) (n = 5). (H) Antibody titres of virus-specific antibodies in the BAL were analysed at day 11 p.i., shown is a titration curve of IgA, IgG1 and IgG2c isotypes (mean ± SEM) (n = 5). Results are representative of at least 2 experiments. The Student’s t test (unpaired) was used: p < 0.05 (*), p < 0.01 (**), p < 0.001 (***), p < 0.0001 (****).

To characterize the adaptive immune response against IAV we again infected p110γ-KD animals with a sub-lethal dose of PR8 IAV and then characterized immune cell infiltration into the BAL and lung at day 7 p.i., which represents the initiation phase of the adaptive immune response. Both CD4+ and CD8+ T cells were readily detected in BAL and lung at this time point (Fig 2A). Quantifying the total number of T cells in the lungs of infected mice it was evident that p110γ-KD mice exhibited a significantly reduced number of both CD4+ and CD8+ cells present (Fig 2B). Most strikingly, nucleoprotein—34-specific (Tet+) CD8+ virus-specific cells were virtually absent in the lungs of p110γ-KD animals. Similarly, the proportion of IFNγ-producing CD4+ and CD8+ cells in the BAL was lower in mice deficient for p110γ kinase function (Fig 2C and 2D). Furthermore, in the lung draining lymph node (dLN) CD4+, CD8+ and virus specific CD8+ T cells were all considerably reduced in the p110γ- defective condition compared to WT animals (Fig 2E). Conversely, no difference was observable in the fold increase in the number of activated CD4+ and CD8+ T cells in the lungs of infected compared to naïve animals (Fig 2F). Similarly, inflammatory cytokines IL-1β and IL-6 in the BAL of infected mice (Fig 2G) were comparable between WT and p110γ-KD mice. In addition, the antiviral B cell response appeared to be completely intact as no difference in antibody titres of different isotypes in the BAL could be observed between WT and p110γ-KD mice (Fig 2H) at day 11 p.i. To address the possibility that the deficient T cell response observed in p110γ-KD mice was due to a T cell defect in naïve animals, T cells were examined in blood and lung in the steady-state. This analysis revealed that the number of T cells in the lung as well as the frequency in the blood of naïve mice was comparable between WT and p110γ-KD animals (S1A and S1B Fig). Furthermore, the frequency of CD44+CD62L- activated T cells was similar regardless of p110γ-deficiency (S1C and S1D Fig). To exclude the possibility that p110γ generally regulates development of hematopoietic cells, the composition of blood of p110γ-KD animals was carefully analysed. Granulocytes such as neutrophils are present at normal levels in p110γ-KD mice (S1E Fig) and similarly the number of red blood cells is also comparable to WT animals in p110γ-KD mice (S1F Fig). Overall, these results suggested an important and specific role for p110γ in regulating the antiviral T cell response, in particular the CD8+ T cell component but that p110γ is largely dispensable for T cell development and activation in the periphery.

### The antiviral T cell response is dependent on the kinase activity of p110γ in the hematopoietic compartment

**Fig 3:**
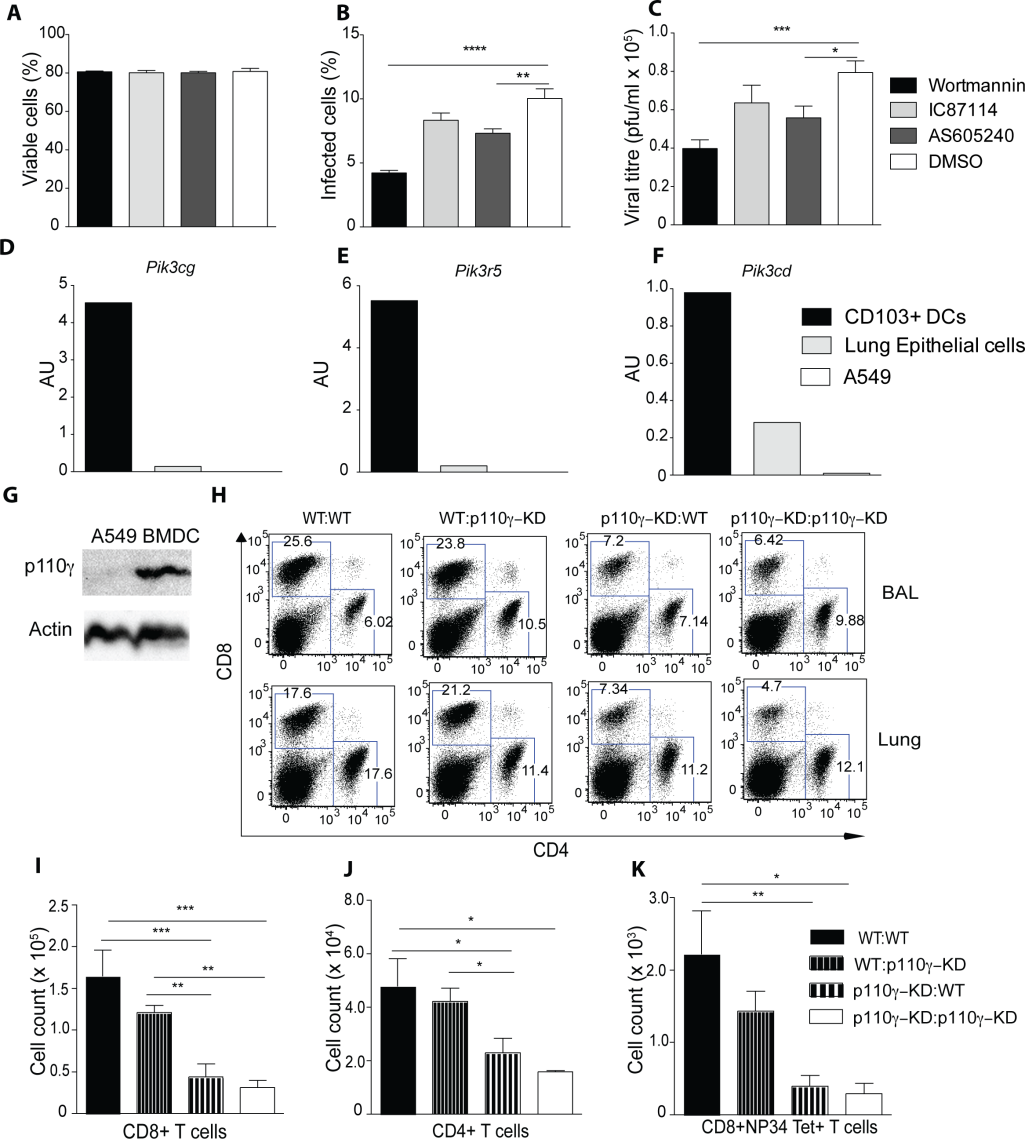
The antiviral T cell response is dependent on p110γ in the hematopoietic compartment. (A-C) A549 cells were treated with inhibitors against p110γ (AS650240), p110d (IC87114), all PI3K subunits (Wortmannin) or DMSO as a control for 30 mins before infecting with PR8 IAV MOI 0.5. Shown is the frequency of viable cells (A) infected cells (B) and the viral titre (C) after 10h of infection (n = 6) (D-F) CD103+ DCs and lung epithelial cells were sorted from WT animals and expression of different PI3K subunits was measured by qPCR, A549 cells were also included. Shown is the expression of Pik3cg, Pik3r5 and Pik3cd normalized to Tbp. Lung CD103+ DCs were identified as CD45+SiglecF-CD11c+MHCII+CD103+CD11b- and lung epithelial cells as CD45-podoplanin+ cells respectively (G) Shown is a western blot of A549 and bone-marrow derived dendritic cells (BMDCs) of p110γ (H-K) WT→WT, WT→ p110γ-KD, p110γ-KD→WT and p110γ-KD→p110γ-KD BM chimeras were generated and subsequently infected with 50 pfu PR8 IAV. The T cell response was analyzed at day 7 p.i. Shown are representative dot plots of CD4+ and CD8+ T cells as well as a summary of all infected animals including NP34 Tet+ CD8+ T cells (mean ± SEM) (n = 5). The results are representative of 2 experiments. One way ANOVA was used with CI 95%: p < 0.05 (*), p < 0.01 (**), p < 0.001 (***), p < 0.0001 (****).

To address a potential direct involvement of p110γ in influenza virus propagation in epithelial cells, A549 cells, a lung epithelial cell line, were infected with PR8 IAV and were also treated with inhibitors against p110γ (i.e. AS605240), p110δ (i.e. IC-87114) or all PI3K subunits (Wormannin). Viability of the cells was not affected by inhibitor treatment (Fig 3A), however the frequency of infected cells as well as the viral titre was significantly reduced in cells treated with Wortmannin, while AS605240 or IC-87114 showed a minor reduction (Fig 3B and 3C). Consistently, p110γ protein was undetectable in A549 cells and mRNA expression of *Pik3cg* and its regulatory subunit *Pik3r5* was barely detectable in sorted lung epithelial cells compared to lung CD103+ DCs (Fig 3D and 3E), while significant expression of another PI3K subunit, *Pik3cd*, could be detected (Fig 3F). To further address the relative importance of p110γ during IAV infection *in vivo* in structural and hematopoietic cells, criss-cross bone marrow chimeras were generated. After reconstitution mice were then infected with IAV. Mice which had received WT bone marrow (BM) mounted a potent anti-viral T cell response, while animals receiving p110γ BM exhibited a significantly reduced number of CD4+, CD8+ and virus specific CD8+ T cells at day 7 p.i. (Fig 3B–3E). Overall, these results suggested that p110γ is required in the hematopoietic compartment for mounting an effective T cell response against IAV.

### p110γ is partially required for proliferation of CD8+ T cells during IAV infection

**Fig 4:**
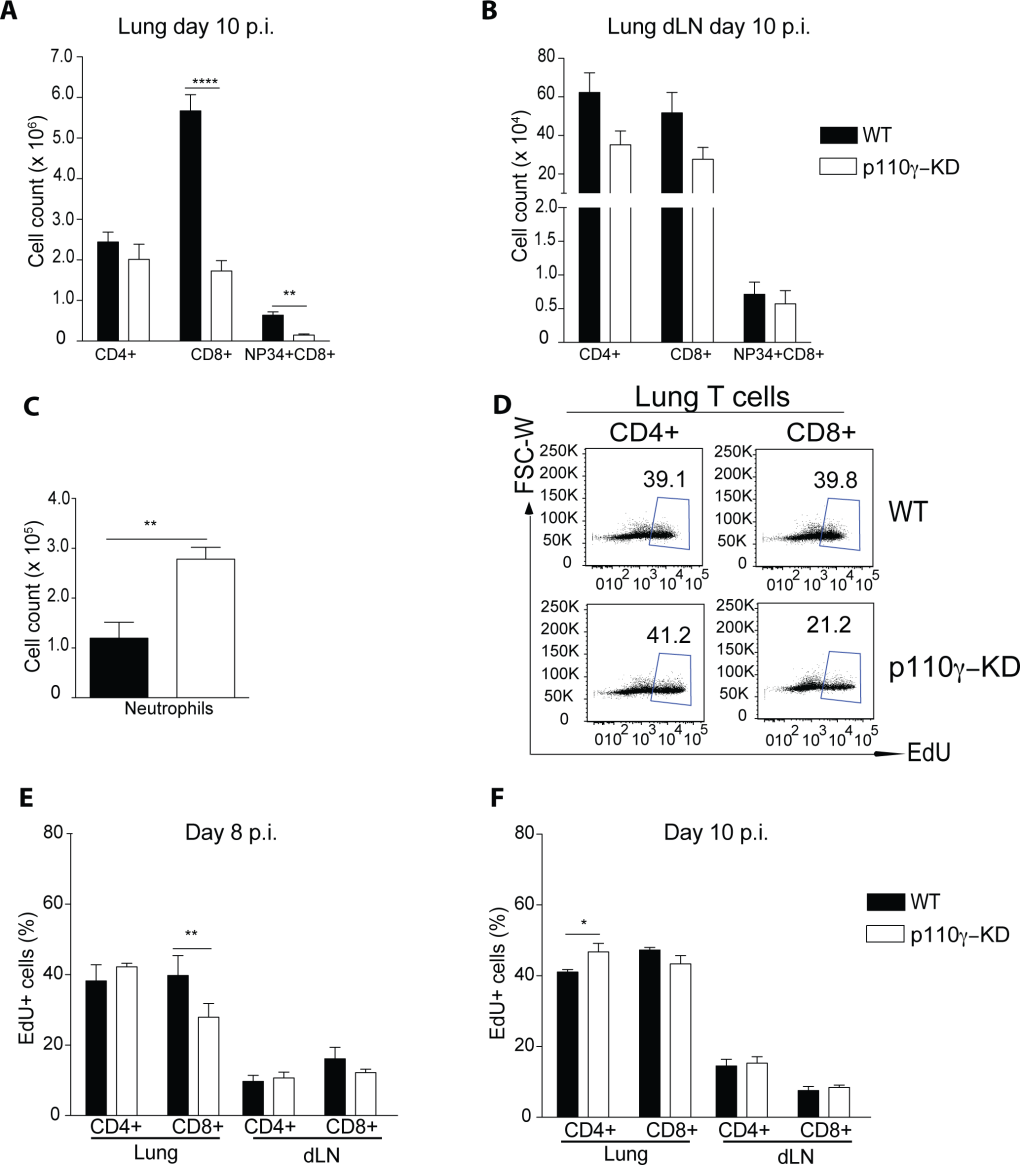
p110γ is largely dispensable for T cell proliferation during IAV infection. WT and p110γ-KD mice were infected with 50 pfu PR8 IAV and the immune cell infiltrate was analysed at day 10 p.i. Cells were pregated on CD45+ viable cells. Shown are (A-B) a summary of T cells in lungs and lung dLN of all mice including NP34 Tet+ CD8+ T cells (mean ± SEM) as well as (C) Neutrophils in the lungs of infected animals (mean ± SEM) (n = 6). At day 6 and day 7 p.i. mice were injected i.p. with EdU and lungs and dLN of infected animals were analyzed on day 8 p.i. to evaluate frequencies of EdU+ cells. Shown are (D) representative dot plots of lung T cells (E) as well as a summary of all infected animals (mean ± SEM) (n = 6). (F) At day 8 and day 9 p.i. mice were injected i.p. with EdU and lungs and dLN of infected animals were analyzed on day 10 p.i. to evaluate frequencies of EdU+ cells. Shown is summary of all infected animals in the lungs and lung dLN (mean ± SEM) (n = 6). The Student’s t test (unpaired) was used: p < 0.05 (*), p < 0.01 (**), p < 0.001 (***), p < 0.0001 (****).

To further dissect the underlying mechanism of the impaired antiviral T cell response in the absence p110γ-kinase function, p110γ-KD mice were infected with a sub-lethal dose of IAV and the T cell response was evaluated at the peak of the response at day 10 p.i. At this time-point the number of CD8+ and Tet+CD8+ T cells in the lung was still strongly reduced in p110γ-KD animals compared to WT controls (Fig 4A). However, CD4+ T cells in p110γ-KD mice were now present in similar numbers to WT animals (Fig 4A). Similarly, no significant differences were visible in the number of CD4+, CD8+ and virus specific Tet+CD8+ T cells in the lung dLN between p110γ-KD and control mice (Fig 4B). In addition, the enhanced morbidity at this time-point post infection of p110γ-KD animals correlated with a more pronounced inflammation in the lung exemplified by a higher number of neutrophils (Fig 4C). To determine whether the reduced number of CD8+ T cells in particular was due to a reduced proliferative capacity of these cells *in vivo*, WT and p110γ-KD mice were infected with IAV and subsequently injected with EdU to measure the proportion of proliferating cells. At an early time-point of the antiviral T cell response the frequency of EdU+ cells among CD8+ T cells but not CD4+ T cells was reduced in p110γ-KD animals (Fig 4D–4E). To shed further light on the kinetics of the T cell response in p110γ-KD mice the same experimental set up was repeated at a later time-point at of infection. During this peak phase of the infection no deficiency in EdU incorporation could be observed for T cells localized in the lungs or dLN of infected p110γ-KD mice compared to controls (Fig 4F). Overall, these results suggested that there is a defect in the priming of CD8+ T cells lacking a functional p110γ kinase domain during IAV infection *in vivo*.

### p110γ is dispensable for T cell priming and migration of dendritic cells *in vitro* and *in vivo*

**Fig 5:**
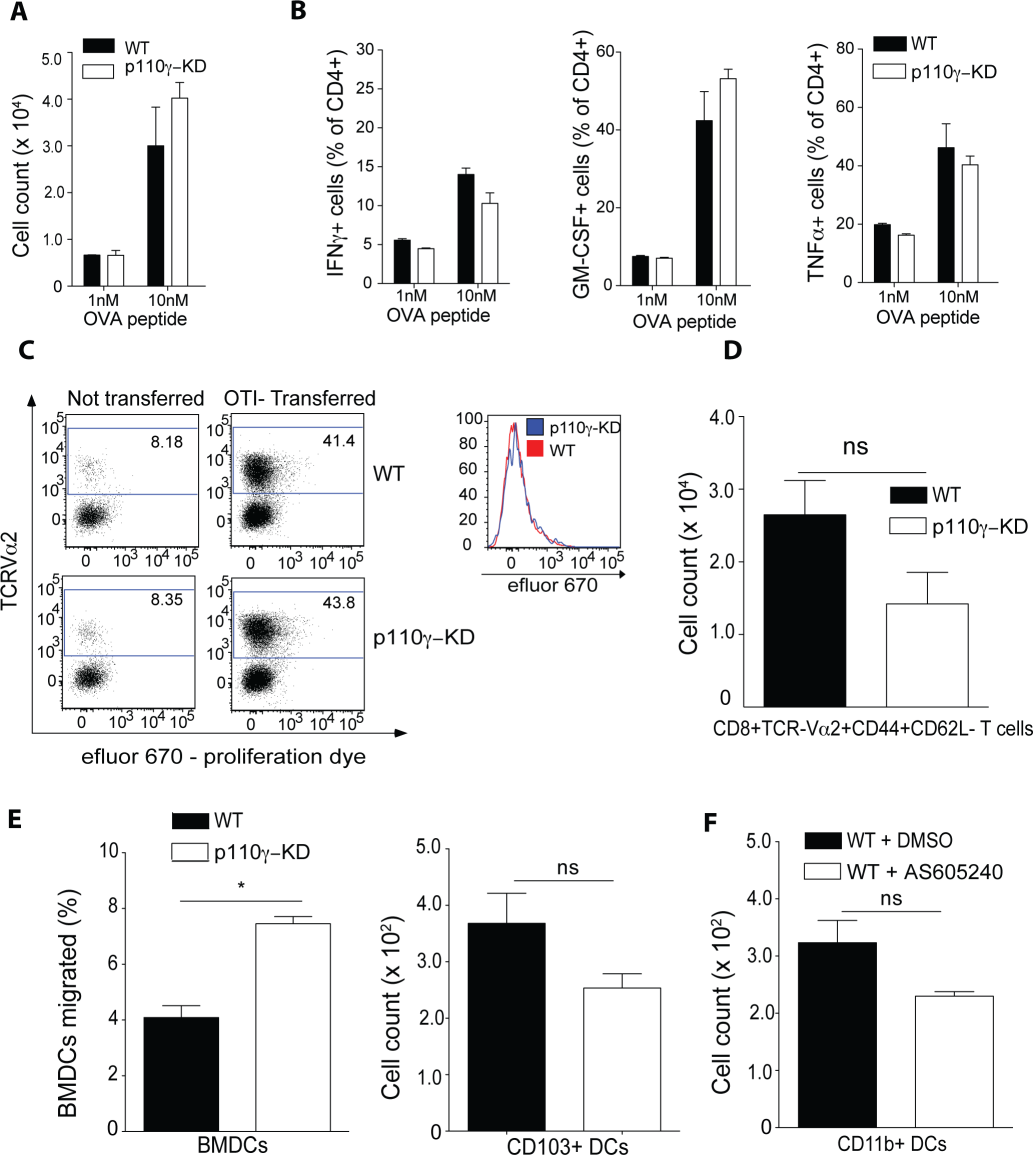
p110γ is dispensable for DC-induced T cell polarization and migration in vitro. WT and p110γ-KD BMDCs were cocultured with OTII CD4+ T cells sorted from the spleen and different concentrations of OVA323-339 peptide for 4 days. Shown are (A) total cell counts of CD4+ T cells (B) as well as production of IFNγ, GM-CSF and TNFα after 4h PMA/ionomcyin restimulation (n = 3). (C-D) 5x106 OT-I cells labeled with efluor 670 were transferred into WT and p110g-KD recipients at day 0. On day 1 after transfer mice received 4mg OVA/Alum into the flank. At day 7p.i. the inguinal lymph nodes were analysed. (C) Shown are representative dot plots of recipients and non-transferred controls as well as a representative histogram overlay of transferred WT and A 6h transwell assay with 500ng/ml CCL21 was conducted with either (C) BMDCs of WT and p110g-KD mice. (D) Shown is the total number of CD8+TCRVa2+CD44+CD62L cells in the inguinal lymph nodes of all analysed animals (mean ± SEM) (n = 5). (E-F) A 6h transwell assay with 500ng/ml CCL21 was conducted with either (E) BMDCs of WT or p110γ-KD origin or (n = 2) (F) dendritic cell subsets sorted from naïve lungs of WT mice (n = 3). (C) Shown is the frequency of migrated cells as summary of all wells (mean ± SEM) (C) or the total number of cells which migrated respectively (D). Lung dendritic cells were sorted as either CD45+Siglec-F-CD11c+MHCII+CD103+CD11b- or CD45+Siglec-F-CD11c+MHCII+CD103-CD11b+CD64- cells representing CD103+ and CD11b+ conventional DCs respectively. Lung DCs were incubated with either 15 μM AS605240 or DMSO as a control. For A-B Ooe way ANOVA was used with CI 95%h. For C-F the student’s t test (unpaired) was used: p < 0.05 (*), p < 0.01 (**), p < 0.001 (***), p < 0.0001 (****).

The T cell response against IAV is strongly dependent on T cell priming by dendritic cells (DC) [31]. To address a potential functional role of p110γ kinase activity in DC-mediated priming of T cells we cocultured bone marrow-derived DCs (BMDCs) of WT and p110γ-KD origin with OTII CD4+ T cells with different concentrations of the OVA_323-339_ peptide. Examining the number of CD4+ T cells after 4 days of culture as a readout for T cell proliferation it was apparent that there is no difference between using WT and p110γ-KD BMDCs (Fig 5A). Furthermore, looking at the T cell polarization in terms of Th1 cytokine production, which is the predominant T helper response during IAV infection, it was evident that p110γ-KD BMDCs have no deficiency in inducing IFNγ, GM-CSF or TNFα producing CD4+ T cells (Fig 5B). To address the possibility that p110γ is required for antigen processing by DCs and that it may play a role in the priming of CD8+ T cells, we transferred efluor-670 labeled OT-I T cells into WT and p110γ-KD recipients and injected ovalbumin in alum one day later. 7 days after transfer we then evaluated the efluor-670 staining as well as the number of TCRVα2+ cells in the inguinal lymph-node, which is the TCRα chain used by all OT-I cells. It was clearly evident that in both WT and p110γ-KD recipients OT-I cells had strongly proliferated as most cells had indeed diluted out the dye completely by day 7 (Fig 5C). Similarly, the number of CD8+CD44+CD62L-TCRVα2+ cells was much higher in the animals, which had received transferred OT-I cells, compared to the non-transferred controls (Fig 5C). Furthermore, there was no statistically significant difference between WT and p110γ-KD recipients. p110γ has been classically associated with regulating migration of immune cells towards chemokines [22] but this has thus far not been thoroughly examined in DCs. CCR7 is a critical regulator of migration of DCs towards the lung dLN and has been shown to be critical for the T cell response against IAV[32]. To test a possible role of p110γ in regulating CCR7-mediated migration of DCs we seeded WT and p110γ-KD BMDCs in a trans-well system and quantified migration towards CCL21, the principal ligand for CCR7. The frequency of migrating BMDCs was higher in p110γ-KD cells compared to WT although generally only 4–8% of cells migrated at all (Fig 5C). To account for the limitations of using BMDCs as a model for lung-resident DCs, a similar experimental set up was repeated using lung DCs. Due to the strong reduction of lung-resident CD103+ DCs in particular in p110γ-KD animals, lung CD103+ and CD11b+ DC subsets were sorted from the lungs of WT animals and then the same migration assay was done in the presence of p110γ-specific inhibitor AS650240 or DMSO as a control. A significant number of both CD103+ and CD11b+ DCs migrated, regardless of p110γ inhibition (Fig 5D). Overall these results suggested that p110γ is not generally required for DC-mediated priming of T cells *in vitro* and *in vivo* as well as for DC migration *in vitro*.

### p110γ is essential for lung DC development in homeostasis and respiratory viral infection

**Fig 6:**
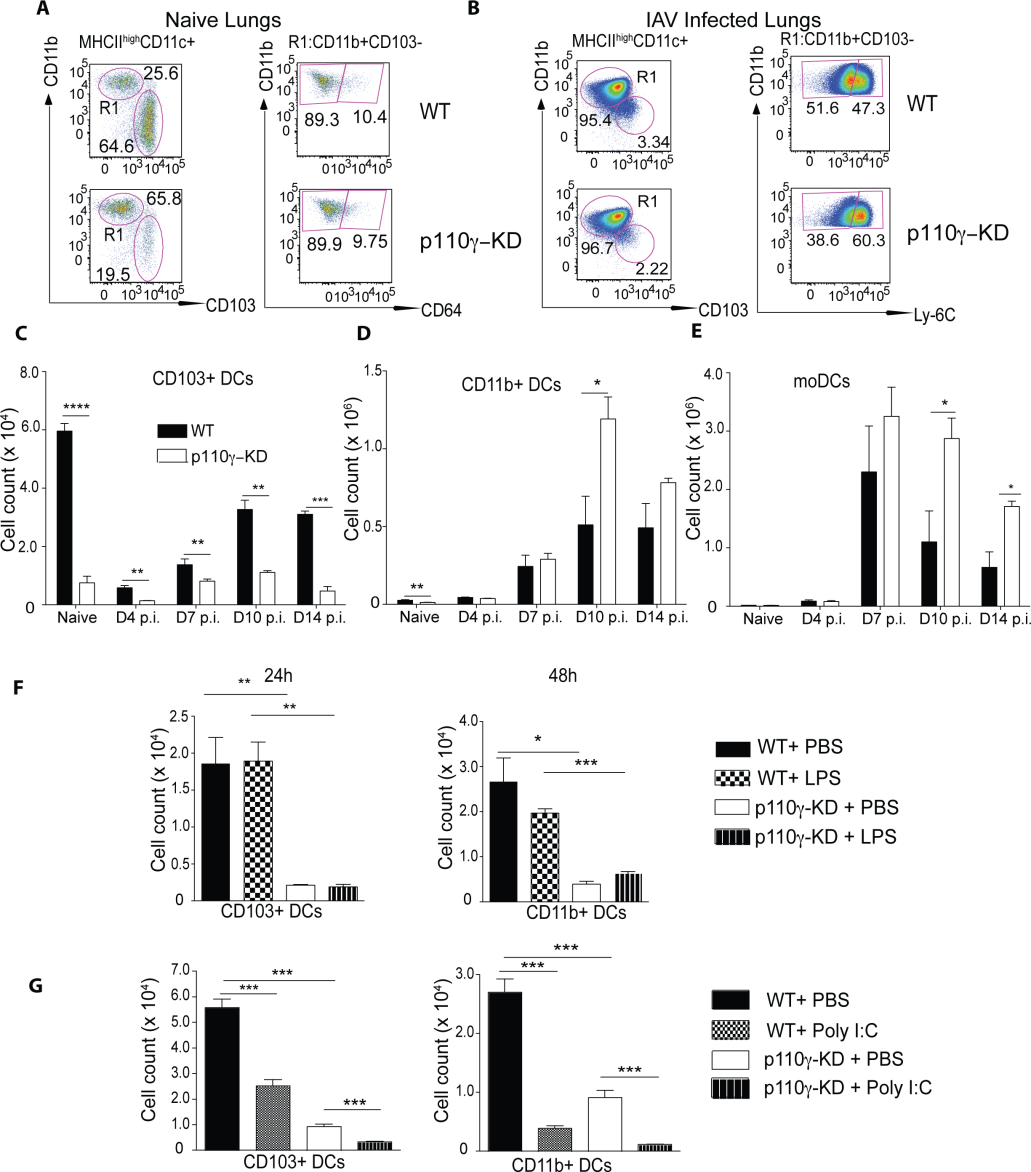
p110γ is essential for lung DC development in homeostasis and respiratory viral infection. WT and p110γ-KD mice were infected with 50 pfu PR8 IAV and the dendritic cell compartment in the lung and BAL was analysed at day 7 and day 10 p.i. Naïve animals are included as a comparison. Shown are (A-B) representative dot plots of naïve and infected lungs as well (C-E) as a summary of infected animals for CD103+ DCs, CD11b+ DCs and moDCs in the lung (n = 4–6) (mean ± SEM). Cells were pregated as CD45+SiglecF-CD11c+MHCII+ viable cells. CD103+ DCs were identified in naïve and infected lungs as CD103+CD11b- cells. CD11b+ DCs were identified as CD11b+CD64- in naïve and CD11b+Ly-6C- in infected lungs and moDCs were identified as CD11b+Ly-6C+ cells in infected lungs and BAL. (F) WT and p110γ-KD mice were inoculated i.t. with 100ng LPS or PBS as a control. Lungs were harvested at 24h or 48h post injection and cellular composition was analysed using flow cytometry. Shown is a summary of FACS dot plots showing the total number of CD103+ dendritic cells (mean ± SEM) (n = 3). (G) WT and p110γ-KD mice were inoculated i.t. with 50ug Poly I:C or PBS as a control. Lungs were harvested at 24h post injection and cellular composition was analysed using flow cytometry. Shown are a summary of FACS dot plots showing the total number of lung CD103+ and CD11b+ dendritic cell subsets (mean ± SEM) (n = 4). The data is representative of at least 2 experiments. The Student’s t test (unpaired) was used: p < 0.05 (*), p < 0.01 (**), p < 0.001 (***), p < 0.0001 (****).

To elucidate the potential role of p110γ in lung DC-mediated antiviral T cell responses *in vivo*, we characterized the lung DC compartment of p110γ-KD mice in the naïve state and during IAV infection. As described by our group recently[23] p110γ-KD animals have a pronounced deficiency in lung-resident conventional DCs in particular the CD103+ subset (Fig 6A, 6C and 6D), while moDCs were present at comparable levels to WT mice in the steady state (Fig 6A and 6D). During IAV infection this picture changed dramatically. moDCs were recruited to the lung in high numbers regardless of p110γ-kinase deficiency (Fig 6B). Conversely, lung CD103+ DCs decreased strongly in number in both WT and p11t0γ-KD animals until day 7 p.i., where the numbers start to increase a later time-points of infection (Fig 6C). For CD11b+ DCs the situation mirrors the kinetics of the CD103+ DCs, although by day 7 p110γ-KD CD11b+ DCs manage to reach numbers similar to WT (Fig 6D). p110γ–deficient CD103+ DCs were always present in lower numbers than WT, although the difference to WT mice became somewhat smaller at the peak of IAV infection (Fig 6C). Overall, these results suggested that the conventional lung-resident CD103+ DCs in p110γ-KD mice are severely deficient at the beginning of an IAV infection and remain so for most of its course, although also in the p110γ–deficient situation the number of CD103+ DCs began to increase again at day 7 p.i. IAV To address the question whether potentially the inflammatory environment can partially overcome the pronounced developmental deficiency of lung-resident DCs in the steady-state in p110γ–deficient animals, we instilled either LPS or PolyI:C intratracheally as a single inflammatory stimulus into the lung and examined how the DC compartment changed over time. Intratracheal injection of a single inflammatory stimulus did not lead to an increase in lung CD103+ DCs in p110γ-KD mice (Fig 6F and 6G). By contrast activation of DCs by these mediators induced a reduction in the cell number, possibly due to their migration do the dLN. Thus the lack of functional p110γ cannot be compensated for by a single inflammatory signal.

### p110γ is crucial for CD103+ DC mediated transport of antigen and apoptotic cells to the draining lymph nodes

**Fig 7:**
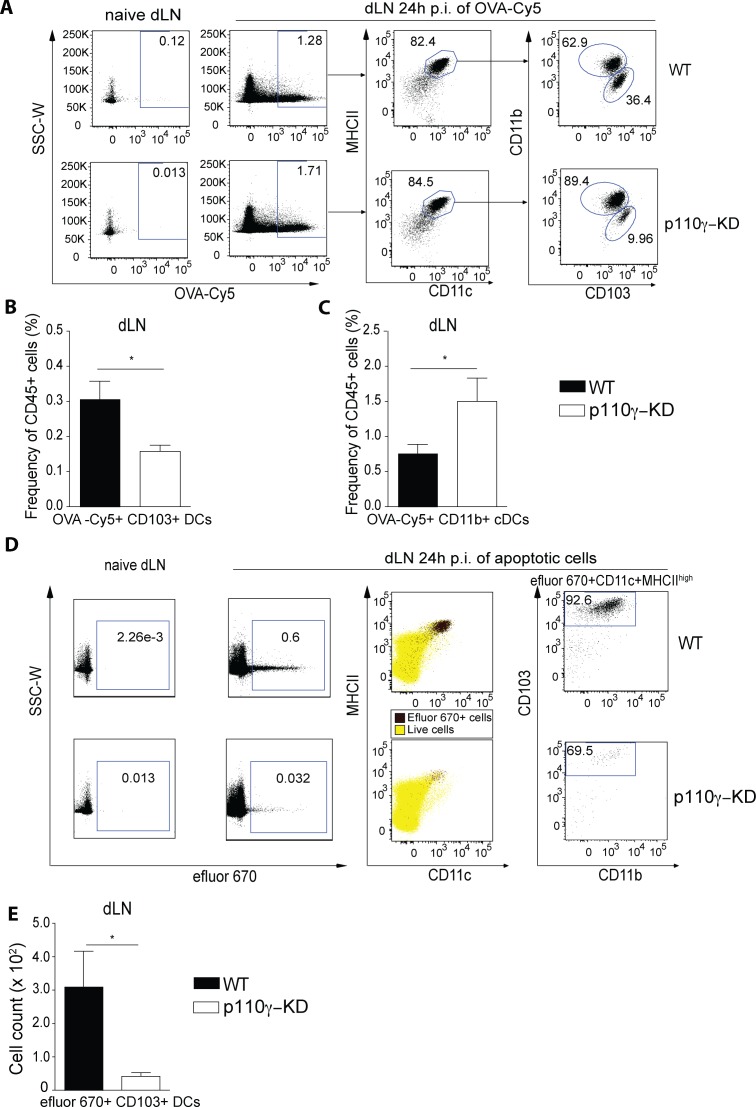
p110γ is required for lung CD103+ DC-mediated transport of antigen to the dLN. (A-C) WT and p110γ-KD mice were injected intratracheally with 40μg OVA-Cy5 and 100ng LPS and sacrificed 24h later for analysis of the lung and the dLN by flow cytometry. DC subsets were identified as CD45+CD11c+Siglec F-MHCIIhigh cells, shown are (A) representative FACS dot plots, as well as a summary of OVA-Cy5+ CD103+ DCs (B) and CD11b+ DCs (C) in the dLN (mean ± SEM) (n = 4), (D-E) p110γ-KD mice were inoculated i.t. with efluor 670 -labeled apoptotic thymocytes or no cells as a control. Lung dLN cells were isolated 24 h later and analyzed by flow cytometry. Efferocytic cells were identified as efluor 670+, and migratory DCs were identified as CD11c+MHCIIhigh. (D) Overlays show efluor 670+ cells (black) and total live cells (yellow) as indicated. (E) Total numbers of efluor 670+ DCs that express CD103 are shown (mean ± SEM) (n = 4). The data is representative of at least 2 experiments. The Student’s t test (unpaired) was used: p < 0.05 (*), p < 0.01 (**), p < 0.001 (***), p < 0.0001 (****).

To address the underlying mechanism of how the absence of p110γ kinase activity in DCs would impact on DC mediated immune responses *in vivo* we administered Cy-5 labeled ovalbumin intratracheally and then observed the migration of DCs to the dLN. After 24 hours cells carrying OVA-Cy5 in the dLN were predominantly DCs in both WT as well as p110γ-KD mice (Fig 7A). However, the proportion of CD103+ DCs carrying OVA in the dLN was considerably reduced in p110γ-KD compared to wild-type mice (Fig 7B). Conversely, the frequency of CD11b+ DCs was somewhat higher in p110γ-KD mice, suggesting a compensatory effect in a situation where pulmonary CD103+ DCs are virtually absent (Fig 7C). To gain a better understanding of the consequences of the strong reduction in pulmonary CD103+ DCs, we sought to evaluate CD103+ DC-specific functions. Lung CD103+ DCs were recently shown to be essential for phagocytosis of apoptotic cells and cross-presentation of antigens in the dLN [33]. To address the efferocytic capacity of pulmonary DCs in p110γ-KD mice we instilled labeled apoptotic thymocytes into the trachea and examined migration of DCs from the lung to the dLN 24h p.i. As previously described, apoptotic cells were almost exclusively transported by CD103+ DCs to the dLN, as opposed to other DC subsets (Fig 7D). Furthermore, while in WT mice a significant amount of CD103+ DCs in the dLN could be found carrying apoptotic thymocytes, these cells were almost completely absent in the dLN of p110γ-KD mice (Fig 7E). Overall, these results suggest that in p110γ-KD animals transport of apoptotic-cell related antigens by cross-presenting DCs is severely deficient.

### p110γ-KD animals are more susceptible to influenza virus infection due to the deficiency in lung CD103+ DCs

**Fig 8:**
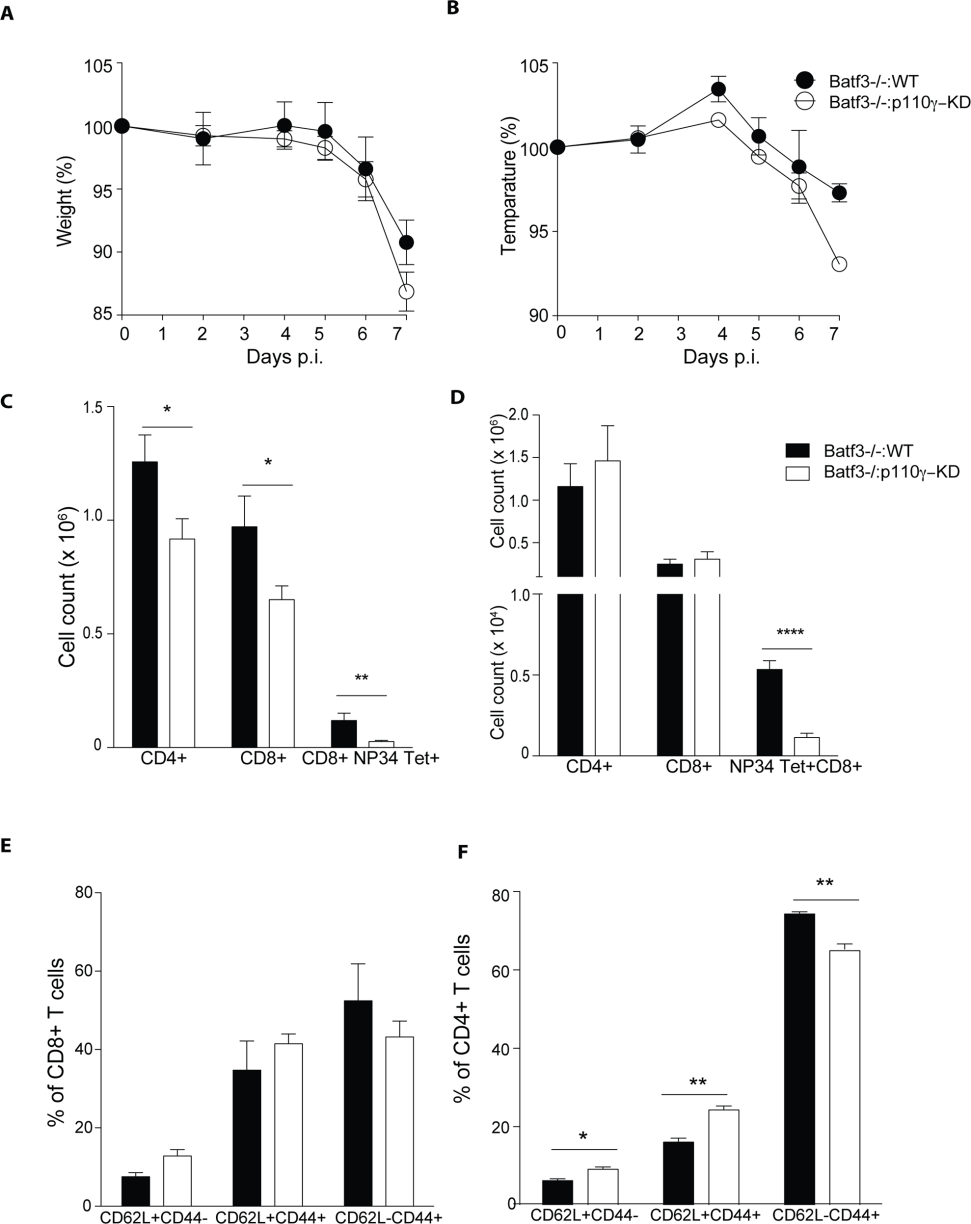
p110γ in lung CD103+ DCs is required for the antiviral CD8+ T cell response. WT mice were irradiated and reconstituted with either Batf3-/-:WT or Batf3-/-:p110γ-KD BM at a ratio of 4:1. 10 weeks after reconstitution mice were infected with 50 pfu PR8 IAV. Shown are (A) the weight and (B) temperature loss during the course of infection. The antiviral T cell response was analysed at day 7 p.i. Shown are a summary FACS dot plots of analysed organs, including the lungs (C) and the lung dLN (D) (mean ± SEM) (n = 5) (E-F) The frequency of CD62L and CD44 expressing T cells in the lungs of infected animals. (mean ± SEM) (n = 5) for all graphs. The Student’s t test (unpaired) was used: p < 0.05 (*), p < 0.01 (**), p < 0.001 (***), p < 0.0001 (****).

To further investigate the notion that a deficiency in cross-presenting lung CD103+ DCs in p110γ-KD animals is responsible for their enhanced susceptibility to IAV infection, we generated BM chimeras using a mixture of either WT or p110γ-KD with Batf3-/- BM at a ratio of 1:4. Batf3 is a transcription factor strictly required for development of lung CD103+ DCs [34]. This led to a situation where at least 80% of T cells were WT, however 100% of lung CD103+ DCs were of either WT or p110γ-KD background. This set up allowed us to address the role of p110γ in lung CD103+ DCs specifically in the context of an IAV infection. Upon Infection animals that had p110γ-deficient CD103+ DCs exhibited more pronounced loss of weight and temperature (Fig 8A and 8B) compared to animals with WT CD103+ DCs. Furthermore, analyzing the T cell response at day 7 p.i. showed that CD4+, CD8+ and virus-specific CD8+ T cells were significantly reduced in the lungs of infected animals (Fig 8C). In lung dLN virus-specific CD8+ T cells were also reduced (Fig 8D). Examining the activation state of T cells in the lung it was evident that the frequency of CD44+CD62L- of CD4+ T cells was reduced in animals, which had received p110γ-KD BM (Fig 8E). However this was not the case for CD8+ T cells (Fig 8F). Overall these results suggested that p110γ in lung CD103+ DCs is required for potent antiviral T cell responses against influenza virus.

## Discussion

In this study we describe PI3Kγ as novel factor, which plays a key role in successful host defense against respiratory infection with influenza virus. We show that the highly increased susceptibility of p110γ-kinase dead animals stems from a defective T cell response leading to higher viral titers and more pronounced morbidity. This phenotype is due to a deficiency in the lung-resident DCs, which are essential for the initial priming of the adaptive immune response against influenza [31]. We could show that PI3Kγ is functionally not required in DCs to activate T cells *in vitro* and *in vivo*, however, the pronounced reduction of lung DCs in naïve PI3Kγ-deficient animals leads to an impaired transport of antigen to the draining lymph node and thus to a defective antiviral T cell response and consequently a delayed viral clearance.

To date the role of PI3Ks in IAV pathogenesis has mainly been analysed from the perspective of the virus, where host PI3Ks were shown to be important for viral replication, in particular of the highly pathogenic PR8 strain [30]. To our knowledge this is the first study reporting an important role of a PI3K family member for the response of the host rather than for viral pathogenesis itself. The well established pro-viral of PI3Ks in general and the novel anti-viral role of PI3Kγ described in this manuscript appear at first conflicting, however, in fact they probably just represent temporally different roles of PI3Ks in the interaction between the virus and its host. PI3Ks have been reported to be important early on during massive viral replication in lung epithelial cells [30] and our results using global inhibition of PI3K signaling support this notion. Furthermore, in this context it appears that PI3Kγ only plays a minor role, which is likely due to its very low expression in lung epithelial cells. The important antiviral role of PI3Kγ only starts to be visible once the PI3Kγ-dependent-DC-mediated T cell response commences with clearance of the virus from the lung. The fact that p110γ-KD mice do not exhibit protection from virus replication earlier on during the infection could reflect that the virus more strongly relies on other PI3K subunits for replication *in vivo*. The virus could also exploit redundancies between the PI3K subunits in epithelial cells, allowing it to continue replicating, despite the functional lack of PI3Kγ. How other PI3K subunits regulate both viral pathogenesis as well as the host immune response, remains to be addressed in future endeavors.

Interestingly, in p110γ-KD animals both CD4+ and CD8+ T cell responses after IAV infection are impaired at an early time-point. The strong defect in CD8+ virus-specific T cells likely stems from the pronounced deficiency in cross-presenting CD103+ DCs in naïve animals, which is similar to that observed in BATF3-/- animals [35]. The minor effect on CD4+ T cells can be explained by the moderate reduction in CD11b+ conventional DCs in the lungs of p110γ-KD animals. This subset has been associated with activating mainly CD4+ T cells [36], although recently it was shown that these cells too can stimulate CD8+ T cells in the context of influenza infection [37]. The deficiency in CD11b+ DCs however, is then overcome by the massive influx of monocyte-derived DCs as well as a novel CD11b+ DCs that likely rescue the CD4+ T cell response at later time-points during the course of the infection. The virus-specific CD8+ T cell response in the lungs of p110γ-KD animals is still strongly deficient by day 10 post infection, underlining the idea that CD103+ DCs are indeed the key players in generating a potent cytotoxic T cell response [31], despite the described capacity of CD11b+ DCs to be able to as well [37]. The deficiency in transport of apoptotic cells observable in p110γ-KD mice fits very well into this picture, as it was recently shown that this capacity was indeed crucial for the ability of CD103+ DCs to cross-present antigen derived from dying cells to CD8+ T cells [37]. Nonetheless, when one examines the frequency of CD8+ T cells which have proliferated in the lungs of infected animals the moderate reduction in EdU+ cells in p110γ-KD animals likely does not completely explain the massive defect in the virus-specific CD8+ T cell compartment. Most probably, lung-resident DCs are not only required for initial priming of the CD8+ T cell response in dLN but also for maintenance of CD8+ effector T cells once they are have reached the pulmonary compartment. This notion has been previously suggested for monocyte-derived DCs [38] and is likely also true for conventional lung-resident DCs. Furthermore, CD4+ T cells proliferate more in p110γ-KD than WT mice at late time-points of infection, which can be likely explained by the continuous presence of virus in the p110γ deficient situation. WT animals have cleared the virus by this stage of the infection and thus antigen is no longer present to stimulate T cell proliferation.

From a developmental point of view the results presented in this paper also bear some novel insights into how DC development changes from steady-state to inflammation. The pronounced developmental organ-specific deficiency of lung-resident CD103+ DCs in the lungs of p110γ-KD animals, which we recently described [23], is partially overcome by the prolonged inflammation during a respiratory viral infection. The numbers of CD103+ DCs decrease strongly until day 4 p.i. but then start to recover at later time-points, also in the p110γ- deficient situation. These results suggest the potential presence of an additional developmental pathway overcoming the requirement for Flt3 signaling in lung CD103+ DC development during inflammation. Possibly this mechanism is also regulated by IL-12 and IFNγ, which has been shown to overcome deficiency of BATF3 in chronic infection with *Mycobacterium tuberculosis* allowing development of lung CD103+ DCs [39]. Unsurprisingly, we could show that for the rerouting of DC development during pulmonary infection, one inflammatory stimulus alone such as LPS or poly I:C in the lung is not sufficient. These observations are also reflected in GM-CSF deficient animals where CD103+ DCs are moderately reduced in adults but this deficiency is then also completely rescued during inflammation [35].

Despite the pre-existing deficiency in lung DCs in p110γ-KD animals, we could also demonstrate that migration of DCs lacking a functional kinase domain of in p110γ is completely normal *in vitro* and *in vivo*, thus showing that p110γ is dispensable for chemokine receptor mediated migration to the dLN. This corrects a notion, which has been suggested for DCs by older publications [40].

Overall our results define PI3Kγ as a new key host factor in the defense against influenza virus infection, establishing a more complex picture about the role of PI3Ks in respiratory viral infection and the interplay between the host and its pathogen.

## Materials and Methods

### Mice

C57BL/6J mice were either bred in-house or purchased from Charles River (Germany). p110γ-KD mice were generated and provided by E. Hirsch, University of Torino and were back-crossed to C57BL/6 for at least 15 generations. BATF3-/- mice were obtained from Jackson (USA). All animals were housed in individually ventilated cages under specific pathogen free conditions at the ETH Phenomics Facility (Zurich, Switzerland) and used for experiments at between 6 and 14 weeks of age unless otherwise stated. The number of mice (n) indicated in the figure legends always refers to the number of animals per group.

### Cells suspension preparations

Mice were euthanized by an overdose of sodium pentobarbital by intraperitoneal injection. Lungs, spleens or lung draining lymph nodes were removed and then processed as described previously [23] Bronchoalveolar lavage was obtained by inserting a catheter into the trachea and subsequently flushing the lungs with PBS for a total volume of 1ml. BAL cells were then obtained by centrifugation and the supernatant was analysed for inflammatory cytokines using ELISA.

### Flow cytometry

Flow cytometry analysis was performed on a FACSCanto II or LSR Fortessa (BD) and analyzed with FlowJo software (Tree Star). Fluorochrome-conjugated or biotinylated monoclonal antibodies specific to mouse CD11c (N418), CD11b (M1/70), Ly-6C (HK1.4), Siglec-F (E50-2440, BD Biosciences), CD103 (2E7), CD45 (30-F11), CD45.1 (A20), CD45.2 (104), CD4 (GK1.5), CD8α (53–6.7), MHC class II (M5/114.15.2, eBioscience), CD64 (X54-5/7.1), CD19 (6D5), CD3e (145-2C11), NK1.1 (PK136, eBioscience), Ly-6G (1A8), podoplanin (8.1.1., eBioscience), Ly-6C (HK1.4), CD49b (DX5), TNF-α (eBioscience) IFN-γ (eBioscience) and GM-CSF (BD) were purchased from Biolegend unless otherwise stated. Dead cells were gated out using the live/dead marker eFluor780 (eBioscience) before analysis. PE-conjugated peptide-MHC class I tetramers (H- 2Db/NP34) with the NP34 peptide (NP366-374; ASNENMETM) from the nucleoprotein of influenza virus A/PR/8/34 were generated as described [41]. Prior to all flow cytometry stainings, FcγIII/II receptors were blocked by incubating cells with homemade anti-CD16/32 (2.4G2).

### Bone marrow chimeras

For bone marrow chimeras, C57BL/6 CD45.2^+^ mice were lethally irradiated (9.5 Gy, using a caesium source) and reconstituted with 5-10x10^6^ BM cells of the background and with the ratio indicated for each experiment. Mice were used for virus experiments weeks post-reconstitution.

### Virus infection

Influenza virus strain PR8 (A/Puerto Rico/34, H1N1) was originally provided by J. Pavlovic, University Zurich. At the age of 6 to 14 weeks, mice were infected intratracheally with varying doses of influenza virus, depending on the experiment. The mice were anaesthetized and inoculated with 50μL virus in endotoxin-free PBS. Temperature and weight of animals was monitored daily and animals were euthanized if they fulfilled severity criteria set out by institutional and cantonal guidelines.

### Viral titer determination

To determine influenza viral titers in the lungs, samples were collected on various days after infection, homogenized (Polytron PT 1300 D), and serially diluted with MDCK cells as described [42].

### Specific restimulation of BAL cells

The day before mice were sacrificed for analysis, 1.5x10^5^ bone marrow-derived dendritic cells (BMDC) [43] were incubated overnight with 1.6x10^5^ pfu UV-inactivated virus (PR8) in 96-well plates. 12h later, these BMDC were pulsed with 1μg/mL NP34 peptide for 2h before BAL cells from individual mice were added. After 2h of incubation at 37°C, Monensin (2μM, Sigma-Aldrich) was added to retain cytokines in the cytoplasm, and cells were again incubated at 37°C for another 3h. Cells were then harvested and stained for flow cytometry analysis.

### Detection of virus-specific antibodies

At the indicated time points, BAL fluid was measured for virus-specific IgA and IgG antibody isotype levels. Ninety-six well plates (Maxisorp; Nunc) were coated with UV-inactivated influenza virus (PR8) in PBS overnight at 4°C. Plates were washed and incubated with PBS-1% BSA for 2h at RT for blocking. BAL fluids from individual mice were serially diluted in PBS-0.1% BSA starting with a 1:2 dilution for BAL fluids and a 1:50 dilution for sera, followed by incubation at RT for 2h. Plates were washed five times and incubated with alkaline-phosphate-labeled goat anti-mouse antibodies to IgG1, IgG2c or IgA (Southern Biotech Technologies, Inc.) at a 1:1000 dilution in PBS-0.1% BSA at RT for 2hrs. Thereafter, plates were washed five times and substrate p-nitrophenyl phosphate (Sigma-Aldrich) was added. Optical densities were measured on an enzyme-linked immunosorbent assay reader (Bucher Biotec) at 405nm.

### OVA-Cy5 and DC migration experiments

For these experiments the Cy5 labeling kit (Axxora) was used with ovalbumin (Invitrogen) to produce OVA-Cy5 according to the manufacturer’s instructions. Mice were anesthetized using isofluorane and injected intratracheally with 40μg OVA-Cy5 and 100ng LPS (Sigma) and tissues were analyzed using flow cytometry one-day post injection.

### Generation of bone marrow-derived dendritic cells

Bone marrow cells were harvested from the femurs of donor animals using a syringe and PBS. Cells were subsequently incubated in complete RPMI-1640 medium (Life Technologies) with 20ng/ml GM-CSF for 7–9 days depending on the experiment. On the day of use non-adherent cells were harvested by gently pipetting and subsequently used for further assays.

### Trans-well assays

DCs were seeded into Costar 5um polycarbonate membrane trans-well plates (Corning) and medium containing 100nM CCL21 was added to the bottom of the well. The plates were incubated for 6h at 37°C and subsequently the migrated cells were harvested and analysed using flow cytometry.

### DC:T cell coculture

BMDCs were obtained as described above. T cells were obtained with CD4 MACS-bead (Miltenyi) sorting from naïve splenocytes. Cells were then cultured together for 4 days in complete IMDM medium (Life Technologies) with the addition of varying concentrations of OVA_323-339_ peptide (Mimotopes Australia). Before flow cytometric analysis, cells were restimulated with PMA (Sigma-Aldrich) ionomycin (Sigma-Aldrich) and monensin (Sigma-Aldrich).

### Administration of LPS and Poly (I:C)

Mice were anesthetized using isofluorane and injected intra-tracheally with either 100ng LPS (Sigma-Aldrich) in PBS or 50μg Poly (I:C) (Invivogen) in PBS respectively. Control mice were just injected with PBS. At the indicated time-points mice were sacrificed and tissues were analyzed using flow cytometry.

### Apoptotic cell delivery

Thymocytes were obtained from C57BL/6 mice and subsequently smashed through a 70um cell strainer to get a single cell suspension. Subsequently apoptosis of these cells was induced by a 120mJ exposure to UV-light. Cells were incubated at 37°C for 2h and were then labeled with efluor 670 (eBioscience) according to the manufacturer’s instructions. After the labeling procedure 1x 10^7^ cells were injected intra-tracheally into recipient mice and tissues were analyzed one day later.

### Inhibitors

A549 cells were treated with inhibitors for 30 min in infection medium (DMEM with 50 mM Hepes and 0.2% BSA, pH 6.8) at a final concentration of 5μM. They were then infected with MOI 0.5 PR8-NS1-GFP virus [44]. 0.2μg/ml TPCK-Trypsin was added and infection was allowed to proceed for 10h. Cells were then analyzed for GFP production using flow cytometry and supernatants were collected for analysis of the virus titre, as described above. The following inhibitors were used: Wortmannin (Sigma), AS605240 (Sigma) and IC87114 (Sigma)

### Western blot

Cells were lysed in laemmli buffer containing 10% β-mercaptoethanol and boiled for 15mins at 95°C. The samples were then separated by SDS-PAGE and transferred to a nitrocellulose membrane. Thereafter the membranes were blocked in 10% milk powder in TBST. Membranes were then incubated in the primary and secondary antibodies at RT for 1h each respectively, with washing in TBST in between. After washing the Supersignal West Pico Chemiluminescent substrate was added and the membranes were imaged on Chemidoc MP imaging system (Biorad). The following antibodies were used: goat anti-mouse p110γ (Santa Cruz Biotechnology), bovine anti-goat HRP (Santa Cruz Biotechnology), goat anti-mouse actin (Santa Cruz Biotechnology).

### Quantitative real-time PCR

For analysis of PI3K subunit expression RNA was isolated from cells with TRIzol reagent (Invitrogen) and was reverse-transcribed with GoScript reverse transcriptase according to the manufacturer’s instructions (Promega). Quantitative real-time RT-PCR was performed with KAPA SYBR FAST. The expression of PI3K subunits was normalized to that of Tbp.

### Statistics

Mean values, SD, SEM, and Student’s t test (unpaired) and One-way ANOVA with CI 95% were calculated using Prism (GraphPad Software, Inc). p < 0.05 (*), p < 0.01 (**), p < 0.001 (***), p < 0.0001 (****).

### Ethics statement

All animal experiments were approved by the local animal ethics committee (Kantonales Veterinärsamt Zürich, licenses ZH270/2014 and 113/2012), and performed according to local guidelines (TschV, Zurich) and the Swiss animal protection law (TschG).

## Supporting Information

S1 Fig p110γ is not required for T cell development in the periphery. Naive WT and p110γ-KD mice were analysed using flow cytometry. Shown is a quantification of T cells in lung (A) and blood (B)(mean ± SEM) (n = 5). (C-D) Shown is the activation state of T cells in naive animals in lung and blood. Activated T cells were defined as CD44+CD62L- cells (mean ± SEM) (n = 5).(E-F) Shown is a quantification of neutrophils and red blood cells in the blood, defined as CD11b+Ly-6G+ and Ter-119+ respectively (mean ± SEM) (n = 5). Results are representative of at least 2 experiments. The Student’s t test (unpaired) was used: p < 0.05 (*), p < 0.01 (**), p < 0.001 (***), p < 0.0001 (****).
(TIF)
