# Supplementary figures and images for: PI3Kγ Is Critical for Dendritic Cell-Mediated CD8+ T Cell Priming and Viral Clearance during Influenza Virus Infection
Source: PLoS Pathog. 2016 Mar 31;12(3):e1005508. doi: 10.1371/journal.ppat.1005508 (PMC4816423; doi:10.1371/journal.ppat.1005508)

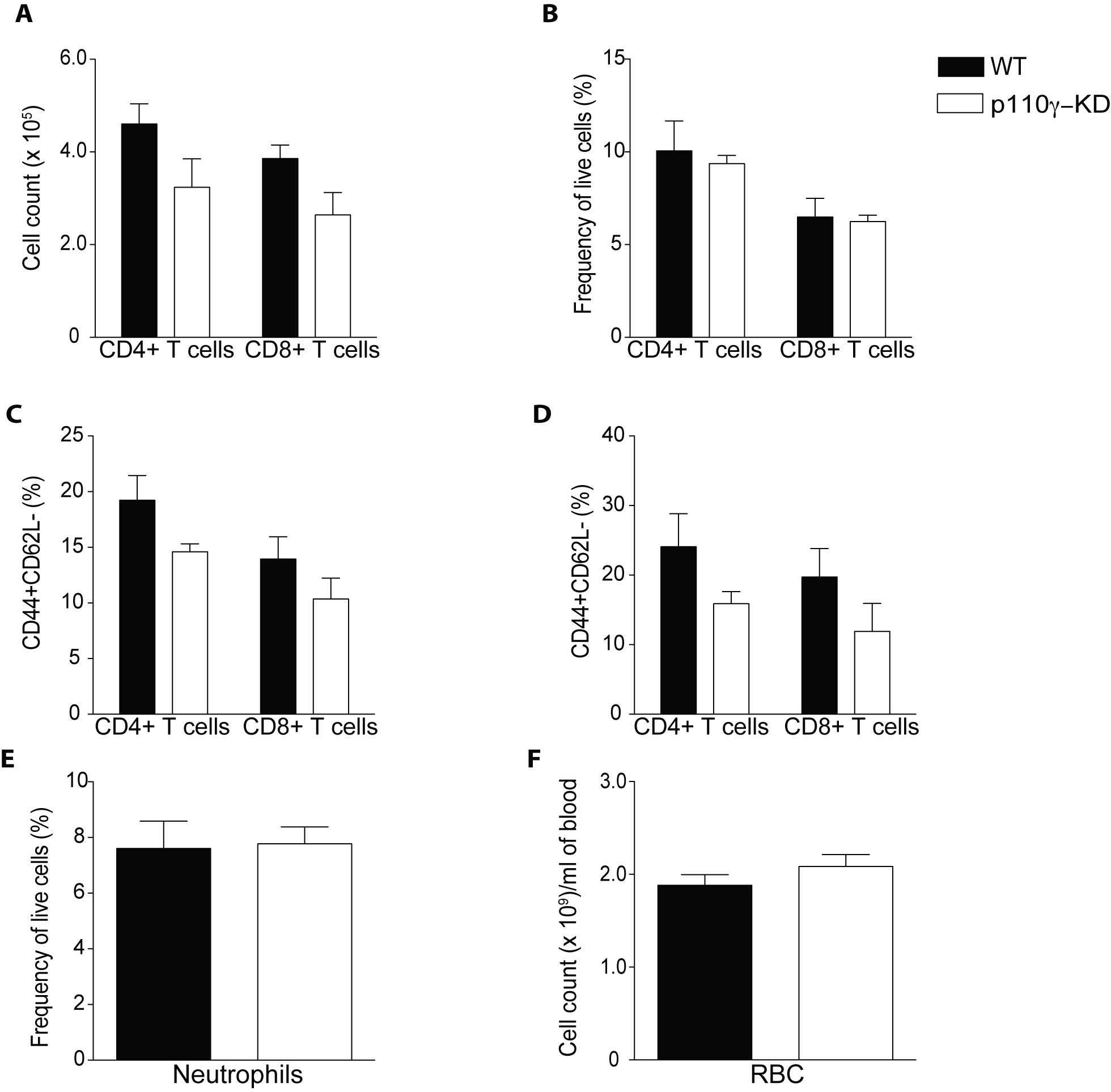

Supplement: S1 Fig — Naive WT and p110γ-KD mice were analysed using flow cytometry. Shown is a quantification of T cells in lung (A) and blood (B)(mean ± SEM) (n = 5). (C-D) Shown is the activation state of T cells in naive animals in lung and blood. Activated T cells were defined as CD44+CD62L- cells (mean ± SEM) (n = 5).(E-F) Shown is a quantification of neutrophils and red blood cells in the blood, defined as CD11b+Ly-6G+ and Ter-119+ respectively (mean ± SEM) (n = 5). Results are representative of at least 2 experiments. The Student’s t test (unpaired) was used: p < 0.05 (*), p < 0.01 (**), p < 0.001 (***), p < 0.0001 (****). (TIF) [file ppat.1005508.s001.tif]
